# Complementary medicine use in the Australian population: Results of a nationally-representative cross-sectional survey

**DOI:** 10.1038/s41598-018-35508-y

**Published:** 2018-11-23

**Authors:** Amie Steel, Erica McIntyre, Joanna Harnett, Hope Foley, Jon Adams, David Sibbritt, Jon Wardle, Jane Frawley

**Affiliations:** 1 Australian Research Centre in Complementary and Integrative Medicine, Faculty of Health, University of Technology Sydney, Ultimo, NSW Australia; 2 Discipline of Public Health, Faculty of Health, University of Technology Sydney, Ultimo, NSW Australia; 3 Endeavour College of Natural Health, Fortitude Valley, Brisbane, QLD Australia; 4 Faculty of Pharmacy, University of Sydney, Sydney, NSW Australia

## Abstract

In order to describe the prevalence and characteristics of complementary medicine (CM) practice and product use by Australians, we conducted a cross-sectional online survey with Australian adults aged 18 and over. Rates of consultation with CM practitioners, and use of CM products and practices were assessed. The sample (n = 2,019) was broadly representative of the Australian population. Prevalence of any CM use was 63.1%, with 36% consulting a CM practitioner and 52.8% using any CM product or practice. Bodywork therapists were the most commonly consulted CM practitioners (massage therapists 20.7%, chiropractors 12.6%, yoga teachers 8.9%) and homeopaths were the least commonly consulted (3.4%). Almost half of respondents (47.8%) used vitamin/mineral supplements, while relaxation techniques/meditation were the most common practice (15.8%). CM users were more likely to be female, have a chronic disease diagnosis, no private health insurance, a higher education level, and not be looking for work. Prevalence of CM use in Australia has remained consistently high, demonstrating that CM is an established part of contemporary health management practices within the general population. It is critical that health policy makers and health care providers acknowledge CM in their attempts to ensure optimal public health and patient outcomes.

## Introduction

The use of complementary medicine (CM), defined as products and practices outside of the dominant medical paradigm^1^, is popular in many parts of the world^2^ and is recognised as a significant public health issue by the World Health Organisation^3^. CM is broadly classified into one of two categories, mind body practices (i.e. yoga, meditation) and natural products (i.e. vitamins, herbal medicines)^1^. The use of CM may be part of a self-management strategy or initiated and monitored under the guidance of a CM practitioner^4^.

The most recent nationally representative investigation of Australian CM use and associated expenditure was conducted over a decade ago^4^. At that time, an estimated 70% of Australians had used at least one form of CM and 44.1% had visited a CM practitioner in the previous 12 months. The number of visits to CM practitioners was almost identical to the estimated number of visits to medical practitioners (69.3 million) and the annual expenditure on CM, was estimated to be 4.13 billion Australian dollars (US $3.12 billion). Well-educated, females aged 18–34 years with a higher than average income were the most common users of CM^4^. As the understanding of CM has evolved since this previous research, there is a need for contemporary data describing CM use to inform current and future health policy and practice in Australia.

It is important to examine the amount of CM currently used in the community, along with the characteristics and drivers of this use, to ensure that appropriate public health policy can be developed to address any risks to the public associated with its use, and any benefits can be incorporated into health service delivery. This will also help to facilitate open discussions with patients about CM use that encourage disclosure and optimise patient care and safety. The objectives of this study are to obtain up-to date data regarding the prevalence and characteristics of CM practice and product use by Australians, and determine the predictors of CM use, using reliable and reproducible instruments and methodology.

## Methods

### Study design and setting

A cross-sectional survey was administered online to an Australian population. Ethical approval for the study was obtained from the Human Research Ethics Committee at Endeavour College of Natural Health (#20170242) in accordance with the Declaration of Helsinki. The survey includes 241 survey items across five survey domains: *demographics*; *health status; health service utilisation; complementary medicine literacy;* and *health information disclosure*. This study represents the first, foundational analysis from the survey data.

### Participants

Participants of this study were Australian adults aged 18 and over (N = 2,025) who were representative of the general population in gender, age and state of residence (Table 1). This study size was determined to provide sufficient statistical power for inferential analysis based upon previous rates of CM use reported in Australian studies^5^.

**Table 1:** Sociodemographic characteristics of survey respondents (n = 2,019) compared with national data from the 2016 National Census^10^.

Characteristics	Survey respondents		National data		*p*
	*n*	%	*n*	%	
**Gender**					
Male	1,034	51.2	10,634,013	49.4	0.10
Female	982	48.6	10,873,704	50.6	
Other	3	0.1	—	—	
**Age (years)**					
18–39	825	40.9	7,714,909	39.2	0.69
40–59	668	33.1	5,975,817	32.3	
60 and over	526	26.1	4,865,978	26.3	
**State**					
New South Wales/Australian Capital Territory	626	31.0	7,274,880	33.8	0.004
Victoria	488	24.2	5,354,042	24.9	
Queensland	464	23.0	4,332,739	20.1	
South Australia/Northern Territory	193	9.6	1,808,517	8.4	
Western Australia	199	9.9	2,239,170	10.4	
Tasmania	49	2.4	495,354	2.3	

### Recruitment

This study used purposive convenience sampling using a database of people who were registered to participate in research. Recruitment and data collection were conducted between 26 July and 28 August, 2017. Respondents who completed the survey received a small financial incentive based on the time taken to complete the survey. The financial incentive is a benefit of being a member of the research company’s (Qualtrics) database. Members of the database who met the inclusion criteria were emailed an invitation to participate in the online survey. Informed consent was obtained once respondents had read the information page presented prior to beginning the survey. The survey took approximately 15 minutes to complete.

Data was screened for disengaged and missing responses. Six respondents were removed as their responses were unreliable (identified by discrepancies between responses, text responses incongruous with the corresponding question, lack of variance, and repeated patterns in the data), leaving 2,019 participants in the final data set.

### Instrument

The survey consisted of 50 items covering demographics, health service utilisation (including use of complementary medicine), health status, health literacy and health communication. The study is a sub-analysis of a larger research project. The measures described below reflect those relevant to the objectives of this study.

### Demographics

Survey items measured gender, age range (categorised in increments of ten years^6^, residential postcode, manageability on household income, highest level of education, private health insurance coverage, health care card status, employment status, and marital status.

### Health service and treatment utilisation

Respondents were asked to provide information about their use of health services and products, including over the previous 12 months. Information about health services use was collected through a survey item which listed both conventional and CM practitioner groups. Details about treatments were measured through a survey item which listed both pharmaceutical (prescription and non-prescription) and non-pharmaceutical treatments. To avoid potential confusion resulting from unclear nomenclature, the terms ‘complementary medicine’ and ‘conventional medicine’ were not used. Items were based on the International Complementary and Alternative Medicine Questionnaire (I-CAM-Q), which was developed as a measure of CM use that could be used consistently across different populations^7,8^.

### Health status

Health status was determined by a single item that asked respondents to describe their general health on a 5-point Likert scale ranging from ‘*excellent’* to ‘*poor’*. Participants were also asked to identify whether they had been diagnosed in the last three years with a health condition from a list of 30 diseases including, but not limited to Australian National Health Priority Area^9^.

### Data analysis

IBM SPSS Statistics Premium Edition Version 22 was used to analyse the data. Relevant variables were recoded to reflect a positive response direction. Binary variables (‘yes, I did use’ or ‘no, I did not use’) were created from categorical variables describing CM use, as well as confounders such as diagnosis with a chronic disease. Descriptive statistics were used to determine frequencies and percentages. Chi-square analysis was used to confirm the representativeness of the sample compared to 2016 Australian Census data^10^, and to test associations between demographic variables and CM use. Backwards stepwise logistic regression was used to control for confounding and determine the most parsimonious model for the likelihood of using any type of CM, any CM treatment, and any CM practitioner. Statistical significance was set at p = 0.05.

## Results

### Participant characteristics

Table 1 summarises the sociodemographic characteristics of participants in comparison to Australian 2016 census data with no differences in age or gender and minor differences in locality; indicating the sample was broadly representative of the Australian population. Table 2 reports the association between any type of CM use and sociodemographic variables. There was a statistically significant association between gender and any CM use; more females (56.2%) compared to males (43.7%) used any type of CM.

**Table 2:** Sociodemographic characteristics of survey respondents.

	All (n = 2019)		Any CM use (n = 1273)		P value
	*n*	%	*n*	%	
**Gender**					<0.001
Female	1,034	51.2	715	56.2	
Male	982	48.6	556	43.7	
Other	3	0.1	2	0.2	
**Age (years)**					0.49
18–29	512	25.4	319	25.1	
30–39	313	15.5	205	16.1	
40–49	362	17.9	235	18.5	
50–59	306	15.2	197	15.5	
60 and over	526	26.1	317	24.9	
**State**					0.86
New South Wales	597	29.6	373	29.3	
Victoria	488	24.2	304	23.9	
Queensland	464	23.0	305	24	
South Australia	188	9.3	118	9.3	
Northern Territory	5	0.2	2	0.2	
Western Australia	199	9.9	121	9.5	
Tasmania	49	2.4	31	2.4	
Australian Capital Territory	29	1.4	19	1.5	
**Employment Status**					<0.001
Full time work	639	31.6	420	33	
Part time work	370	18.3	251	19.7	
Casual/temp work	139	6.9	92	7.2	
Looking for work	185	9.2	90	7.1	
Not in the paid workforce	686	34.0	420	33	
**Marital status**					0.02
Never married	584	28.9	352	27.7	
Married	864	42.8	564	44.3	
De facto (opposite sex)	220	10.9	125	9.8	
De facto (same sex)	29	1.4	23	1.8	
Separated/Divorced/Widowed	322	16.0	209	16.4	
**Highest qualification**					<0.001
Less than Year 12	327	16.2	178	14	
Year 12 or equivalent	421	20.9	231	18.1	
Trade/apprenticeship/certificate/diploma	682	33.8	444	34.9	
University degree	589	29.1	420	33	
**General Health Status**					0.44
Poor	164	8.4	111	8.7	
Fair	440	21.8	271	21.3	
Good	643	31.8	415	32.6	
Very good	598	29.6	373	29.3	
Excellent	174	8.6	103	8.1	
**Chronic disease diagnosis**	1314	65.1	897	70.5	<0.001
**Financial management**					0.61
It is impossible/It is difficult all of the time	430	21.3	260	20.4	
It is difficult some of the time	766	37.9	486	38.2	
It is not too bad	700	34.7	446	35	
It is easy	123	6.1	81	6.4	
**Contribution to costs of health care**					
Health care card	839	41.6	542	42.6	0.22
Private health insurance (PHI)	1028	50.9	576	45.2	<0.001

### Prevalence and frequency of health service and treatment use

A total of 1,273 people (63.1%) used CM (either treatments or consulted practitioners), with 36% consulting with at least one CM practitioner and 52.8% using any CM product or practice. Bodywork therapists such as massage therapists, chiropractors and yoga teachers were the most commonly consulted CM practitioner (Table 3). The prevalence rate of consultations with an acupuncturist (7.9%), naturopath (6.2%), osteopath (5.4%) and TCM practitioner (5.3%) was comparable to each other. The prevalence rate for vitamin/mineral supplements use was the highest of all CM products (47.8%), while relaxation techniques or meditation was the most frequently reported CM practice (15.8%).

**Table 3:** Prevalence and frequency of conventional and CM health service and treatment utilization.

Conventional healthcare	*n*	(%)	Complementary medicine	*n*	(%)	
**Medical doctor**			**CM Practitioner**			
General practitioner	1,756	87.0	Massage therapist	418	20.7	
Specialist doctor	839	41.6	Chiropractor	254	12.6	
Hospital doctor	568	28.1	Yoga teacher	180	8.9	
**Allied health**			Acupuncturist	159	7.9	
Pharmacist	1,544	76.5	Naturopath	126	6.2	
Physiotherapist	435	21.5	Osteopath	110	5.4	
Counsellor/psychologist	418	20.7	TCM practitioner	107	5.3	
Community nurse	204	10.1	Aromatherapist	79	3.9	
Other allied health	24	1.2	Western herbalist	76	3.8	
**Pharmaceuticals**			Homeopath	68	3.4	
Prescription-only	1,502	74.4	Other natural health practitioner	9	0.4	
Over-the-counter	1,349	66.8	Traditional/Spiritual healer	2	0.1	
			***Any CM practitioner***	726	36.0	
			**CM Products and Practices**			
			Vitamin/mineral supplements	966	47.8	
			Aromatherapy oils	224	11.1	
			Western or Chinese herbal medicines	191	9.5	
			Flower essences	151	7.5	
			Homeopathy	138	6.8	
			***Any CM product***	1,016	50.3	
			Relaxation techniques/meditation	320	15.8	
			Yoga, Tai Chi or Qi Gong	237	11.7	
			***Any CM Practice***	377	18.7	
			***Any CM Product or Practice***	1,067	52.8	

### Characteristics of complementary medicine users

Table 4 reports the results of the logistic regression models predicting the likelihood of each type of CM use. The model assessing the likelihood of demographic characteristics associated with any CM use (practitioner or treatment), was statistically significant, χ^2^(13) = 170.04, p < 0.001, and correctly classified 65.6% of cases. People without a chronic disease diagnosis, were less likely to use any type of CM (OR 0.49; p < 0.001). Those without private health insurance were more likely to use any type of CM compared to those with health insurance (OR 1.71; p < 0.001). Females were more likely than males to use any type of CM (OR 1.78; p < 0.001). There was a significant effect for education (p < 0.001); those with either a less than year 12, or year 12 or equivalent qualification were less likely to use any type of CM compared to those with a university degree. Employment status also had a significant influence on the model p = 0.003); full-time or part-time employment was more likely to influence any type of CM use, and those who were looking for work were less likely to use any type of CM compared to those not in the paid workforce.

**Table 4:** Logistic regression predicting characteristics of individuals who use CM treatments (products or practices), consult with CM practitioners, and use any type of CM.

Characteristics	Any type of CM (practitioner or treatment) use			CM practitioner consultations			CM treatment (products and practices) use		
	*Odds ratio*	*95% CI*	*p value*	*Odds ratio*	*95% CI*	*p value*	*Odds ratio*	*95% CI*	*p value*
**Gender (female)**	1.78	1.46–2.16	<0.001	1.59	1.30–1.94	<0.001	1.78	1.48–2.14	<0.001
**Employment status**			0.003			<0.001			0.09
Full time work	1.29	1.00–1.67	0.05	2.63	2.02–3.41	<0.001	0.94	0.73–1.19	0.59
Part time work	1.33	1.00–1.77	0.05	1.96	1.47–2.61	<0.001	1.12	0.85–1.47	0.42
Casual/temp work	1.34	0.88–2.02	0.16	1.72	1.15–2.58	0.01	1.12	0.76–1.66	0.56
Looking for work	0.70	0.49–0.99	0.04	0.80	0.53–1.21	0.29	0.68	0.48–0.96	0.03
Not in the paid workforce	Ref	—	—	Ref	—	—	Ref	—	—
**Highest level of qualification**			<0.001			0.04			<0.001
Less than Year 12	0.59	0.43–0.81	0.001	0.68	0.49–0.94	0.02	0.57	0.42–0.77	<0.001
Year 12 or equivalent	0.58	0.43–0.77	<0.001	0.70	0.53–0.93	0.01	0.53	0.40–0.70	<0.001
Trade/apprenticeship/certificate/diploma	0.87	0.67–1.12	0.27	0.83	0.65–1.07	0.15	0.78	0.61–1.00	0.05
University degree	Ref	—	—	Ref	—	—	Ref	—	—
**Chronic disease diagnosis (no)**	0.49	0.40–0.60	<0.001	0.57	0.46–0.70	<0.001	0.50	0.41–0.61	<0.001
**Private health insurance (no)**	1.73	1.42–2.12	<0.001	1.69	1.38–2.08	<0.001	1.39	1.15–1.69	0.001

The second logistic regression assessing the likelihood of consulting with a CM practitioner was statistically significant, χ^2^(14) = 190.81, p < 0.001, and correctly classified 67.7% of cases. Females were more likely to consult with a CM practitioner compared to males (OR 1.59, p < 0.001). People without a chronic disease diagnosis compared to those with a diagnosis, were less likely to consult with a CM practitioner (OR 0.57, p < 0.001). Those without private health insurance compared to those with health insurance were more likely to consult with a CM practitioner (OR 1.69, p < 0.001). Qualification has a significant effect on the model (p = 0.04); those with a qualification of either less than year 12, year 12 or equivalent were less likely to consult with CM practitioners compared to those with a university degree. All types of employment were more likely to influence any type of CM use compared to those not in the paid workforce, except looking for work p < 0.001). People in full time employment were 2.63 times more likely to consult with a CM practitioner.

Finally, the logistic regression testing the likelihood of using any CM treatment (product or practice) was statistically significant, χ^2^(13) = 149.41, p < 0.001, and correctly classified 61.3% of cases. Females were more likely than males to use a CM treatment (OR 1.78, p < 0.001). People without a chronic disease diagnosis, were less likely to use a CM treatment compared to those with a diagnosis (OR 0.50, p < 0.001). In contrast, those without private health insurance were more likely to use a CM treatment compared to those with private health insurance (OR 1.39, p = 0.001). Level of qualification had a statistically significant influence on the model (p < 0.001); all levels of qualification were less likely to influence consultations with CM practitioners, compared to a university degree. Employment status also had a significant effect; people who were looking for work were less likely to use CM treatments compared to those not in the paid workforce (p = 0.03).

## Discussion

Our study represents the first comprehensive examination of CM use by the Australian population using a nationally-representative survey for over a decade. The findings of this study suggest that two out of three Australians use some form of CM. This figure is consistent with previous studies indicating that high levels of CM use^4^ are a firmly entrenched aspect of the healthcare milieu in Australia, with prevalence and utilisation levels that are both significant and consistent.

This study is not without limitations. The self-reported nature of the surveys exposes the data to the risk of responder and recall bias. However, previous research suggests this may not be such an issue for recall of events over a 12 month period particularly for recall of symptoms and conditions^11^. As the only items in our study where respondents were asked to recall health events which extended beyond a 12 month period related to diagnosis with or treatment for a chronic health condition (in the previous three years) it is not expected that this will have significantly affected the integrity of the survey data. Our data were also unable to provide insights into the health outcomes of CM. Despite these limitations, the nationally-representative sample importantly affords generalisability to the findings and as such this study has potential value to Australian policy makers, researchers and health professionals.

Individuals in our study with a chronic disease diagnosis were more likely to use CM compared with the general population; a finding which was consistent across all categories of CM use examined. This finding concurs with previous Australian studies in discrete populations which suggested higher rates of CM utilisation among those with chronic or co-morbid conditions^5^. Chronic disease is a high priority in Australian health policy due to the substantive burden of disease and the associated complexity of health service needs for individuals with diagnosed chronic health complaints^12^. Persons with chronic conditions are known to be higher users of healthcare services more generally, and as such higher CM use may simply be a reflection of this broader phenomenon. However, it is also possible that people with chronic conditions are not having their healthcare needs met by conventional services and treatments, and are seeking CM use to supplement conventional care and address their unmet needs. In any case, the higher utilisation of CM by persons with increasingly complex health conditions and potential co-morbidities—combined with the fact that these patients are more likely to use a variety of healthcare approaches—presents a number of risks to the CM user^13^. These risks need to be managed both clinically (e.g. by ensuring medical providers actively inquire as to their patient’s CM use, as patients are more likely than not to be using some form of CM) and through appropriate policy (e.g. by ensuring appropriate levels of regulation).

The finding that those *without* PHI were significantly more likely to use any type of CM, consult a CM practitioner, and use a CM treatment conflicts with findings from previous studies^5^. This finding is interesting because other factors that are traditionally associated with higher uptake of PHI – such as gender, income and education – are also associated with higher utilisation of CM^5^. However, many of these previous studies focused on whether PHI users specifically had ancillary cover – cover specific for CM and additional allied health services – which represents only a minority of policies^14,15^. This suggests that CM may not be a primary driver for PHI, which in Australia is focused largely on hospital coverage. This is further supported by findings when other therapies are similarly analysed, with higher use only found for specific policies rather than PHI more broadly (e.g. dental)^15^. Lack of uptake of CM-specific insurance may also indicate that CM users may not see value in PHI plans, which may be restrictive or incomplete (for example, PHI does not cover CM prescriptions), and prefer to use their discretionary healthcare spend to self-insure for these services rather than utilise PHI. Although the interface between CM and PHI has been the topic of considerable discussion^16^, findings such as these indicate that greater clarity is required to appropriately inform policy in this area.

Our study found one third of Australians had consulted with a CM practitioner in the previous 12 months; a finding supported by previous research^4^. CM practitioners consulted most frequently appear to be body work and manual therapists such as massage therapist and chiropractors, confirming previous research. Practitioners for these health professions may be contributing significantly to the non-pharmacologic management of conditions such as back pain^17,18^, treatment options which have received additional attention where conventional approaches such as opioids have received increasing attention for uncertain effectiveness and safety^19^. The clearly articulated role for these CM professions for a limited range of conditions may partly explain these higher levels of utilisation. The use of these therapies for musculoskeletal conditions—but not other conditions—attracts a high level of support for referring practitioners as well as attracting a small number of subsidies for their services^20,21^. CM professions such as acupuncture and naturopathy, who report a broader scope of conditions treated^22^, were also identified as being used by a notable number of Australians in our study. While there is growing evidence suggesting effectiveness for acupuncture^23–26^ and for naturopathic care^27,28^ in a range of clinically important health conditions, lack of integration and variability in standards, regulation and research attention on these CM professions means that the impact these practitioners have on healthcare delivery remains largely unknown. Equally any difference in health outcomes from CM products or treatments when delivered by registered rather than unregistered health professionals have also been overlooked in the research to date. Ultimately, more research that explores the interface, interaction and effects of CM on conventional health care, and conventional health care on CM, is needed.

## Conclusion

CM use in Australia has remained consistently high over the last decade despite ongoing developments in the evidence and policy environment surrounding health care utilisation. Consequently, it is critical that researchers, health policy makers and providers consider CM in relation to their investigations, interventions and attempts to provide optimal public health and patient care.

## Data Availability

The datasets generated during and analysed during the current study are available from the corresponding author on reasonable request.

## References

[1] U. S. Department of Health and Human Services. *Collection Development Manual of the National Library of Medicine* (ed. National Institutes of Health) (National Library of Medicine, National Institutes of Health, U.S. Department of Health and Human Services, Bethesda, MD, 2004).

[2] Coulter ID, Willis EM (2004). The rise and rise of complementary and alternative medicine: a sociological perspective. Med. J. Aust..

[3] World Health Organisation. *WHO Traditional Medicine Stategy 2014*–*2023*, http://www.who.int/medicines/publications/traditional/trm_strategy14_23/en/ (2016).

[4] Xue Charlie C.L., Zhang Anthony L., Lin Vivian, Da Costa Cliff, Story David F. (2007). Complementary and Alternative Medicine Use in Australia: A National Population-Based Survey. The Journal of Alternative and Complementary Medicine.

[5] Reid R, Steel A, Wardle J, Trubody A, Adams J (2016). Complementary medicine use by the Australian population: a critical mixed studies systematic review of utilisation, perceptions and factors associated with use. BMC Complement. Altern. Med..

[6] Turner EL, Dobson JE, Pocock SJ (2010). Categorisation of continuous risk factors in epidemiological publications: a survey of current practice. Epidemiol. Perspect. Innov..

[7] Quandt SA (2009). Development of an international questionnaire to measure use of complementary and alternative medicine (I-CAM-Q). J. Altern. Complement. Med..

[8] Quandt SA, Ip EH, Saldana S, Arcury TA (2012). Comparing two questionnaires for eliciting CAM use in a multi-ethnic US population of older adults. Eur. J. Integr. Med..

[9] National Health and Medical Research Council. *NHMRC Corporate* Plan *2016–2017* (ed. National Health and Medical Research Council) (Australian Government, Canberra, ACT, 2016).

[10] Australian Bureau of Statistics. *2016 Census QuickStats*, http://quickstats.censusdata.abs.gov.au/census_services/getproduct/census/2016/quickstat/036 (2017).

[11] Navin CT, Stewart WJ, Parkinson L, Sibbritt D, Byles J (2015). Identification of diabetes, heart disease, hypertension and stroke in mid-and older-aged women: comparing self-report and administrative hospital data records. Geriatr. Gerontol. Int..

[12] Australian Health Ministers Advisory Council. *National Strategic Framework for Chronic Conditions*. (ed. Australian Government Department of Health) (Australian Government, Canberra, 2017).

[13] Wardle J, Adams J (2014). Indirect and non-health risks associated with complementary and alternative medicine use: an integrative review. Eur. J. Integr. Med..

[14] Spinks J, Hollingsworth B (2012). Policy implications of complementary and alternative medicine use in Australia: data from the National Health Survey. J. Altern. Complement. Med..

[15] Gnanamanickam ES, Teusner DN, Arrow PG, Brennan DS (2017). Dental insurance, service use and health outcomes in Australia: a systematic review. Australian Dental Journal.

[16] Wardle, J. The review of natural therapies for private health insurance: what does it say and what does it mean? *Adv. Integr. Med*. **3** (2016).

[17] Murthy V (2014). Consultations with complementary and alternative medicine practitioners amongst wider care options for back pain: a study of a nationally representative sample of 1,310 Australian women aged 60–65 years. Clin. Rheumatol..

[18] Steel A (2012). Utilisation of complementary and alternative medicine (CAM) practitioners within maternity care provision: results from a nationally representative cohort study of 1,835 pregnant women. BMC Pregnancy Childbirth..

[19] Deyo RA, Von Korff M, Duhrkoop D (2015). Opioids for low back pain. BMJ..

[20] Wardle J, Sibbritt D, Adams J (2013). Referrals to chiropractors and osteopaths: a survey of general practitioners in rural and regional New South Wales, Australia. Chiropr. Man. Ther..

[21] Wardle JL, Sibbritt DW, Adams J (2013). Referral to massage therapy in primary health care: a survey of medical general practitioners in rural and regional New South Wales, Australia. J. Manipulative Physiol. Ther..

[22] Steel, A. *et al*. The Australian complementary medicine workforce: a profile of 1,306 practitioners from the PRACI study. *J. Altern. Complement. Med*. **In Press** (2017). 10.1089/acm.2017.0206 29293360

[23] Pilkington K, Kirkwood G, Rampes H, Cummings M, Richardson J (2007). Acupuncture for anxiety and anxiety disorders – a systematic literature review. Acupunct. Med..

[24] Griggs C, Jensen J (2006). Effectiveness of acupuncture for migraine: critical literature review. J. Adv. Nurs..

[25] White AR (2003). A review of controlled trials of acupuncture for women’s reproductive health care. J. Fam. Plann. Reprod. Health Care..

[26] Hutchinson AJP, Ball S, Andrews JCH, Jones GG (2012). The effectiveness of acupuncture in treating chronic non-specific low back pain: a systematic review of the literature. J. Orthop. Surg. Res..

[27] Arentz S, Abbott JA, Smith CA, Bensoussan A (2014). Herbal medicine for the management of polycystic ovary syndrome (PCOS) and associated oligo/amenorrhoea and hyperandrogenism; a review of the laboratory evidence for effects with corroborative clinical findings. BMC Complement. Altern. Med..

[28] Oberg EB (2015). Estimated effects of whole-system naturopathic medicine in select chronic disease conditions: a systematic review. Altern. Integr. Med..

